# Updated confidence intervals for the COVID-19 antibody retention rate in the Korean population

**DOI:** 10.5808/GI.2020.18.4.e45

**Published:** 2020-12-24

**Authors:** Md. Kamruzzaman, Catherine Apio, Taesung Park

**Affiliations:** 1 Department of Statistics, Seoul National University, Seoul 08826, Korea; 2 Interdisplinary Program in Bioinformatics, Department of Statistics, Seoul National University, Seoul 08826, Korea

**Keywords:** confidence interval, COVID-19 antibody, retention rate

## Abstract

With the ongoing rise of coronavirus disease 2019 (COVID-19) pandemic across the globe, interests in COVID-19 antibody testing, also known as a serology test has grown, as a way to measure how far the infection has spread in the population and to identify individuals who may be immune. Recently, many countries reported their population based antibody titer study results. South Korea recently reported their third antibody formation rate, where it divided the study between the general population and the young male youths in their early twenties. As previously stated, these simple point estimates may be misinterpreted without proper estimation of standard error and confidence intervals. In this article, we provide an updated 95% confidence intervals for COVID-19 antibody formation rate for the Korean population using asymptotic, exact and Bayesian statistical estimation methods. As before, we found that the Wald method gives the narrowest interval among all asymptotic methods whereas mid p-value gives the narrowest among all exact methods and Jeffrey’s method gives the narrowest from Bayesian method. The most conservative 95% confidence interval estimation shows that as of 00:00 November 23, 2020, at least 69,524 people were infected but not confirmed. It also shows that more positive cases were found among the young male in their twenties (0.22%), three times that of the general public (0.051%). This thereby calls for the quarantine authorities’ need to strengthen quarantine managements for the early twenties in order to find the hidden infected people in the population.

## Introduction

In October, US President Donald J. Trump received Regeneron Pharmaceuticals’ experimental monoclonal antibody (mAb) cocktail REGN-COV2 as part of his treatment for coronavirus disease 2019 (COVID-19) when diagnosed with the novel severe acute respiratory syndrome coronavirus 2 (SARS-CoV-2) on October 2, 2020. These and 11 other experimental mAb treatments targeting the SARS-CoV-2 S protein are undergoing human testing, with at least another 150 other antibodies in discovery research [1].

As the COVID-19 pandemic continues to unfold, interests has grown in COVID-19 antibody testing, also known as a serology test, as a way to measure how far the infection has spread in the population and to identify individuals who may be immune [2]. The antibody titer test is a test that checks whether antibodies have been formed in the body after an infection with COVID-19. Antibodies are proteins usually formed in the body by the immune system in response to an infectious disease. Therefore, if these antibodies are tested, the total sample size of patients, including those who passed by without knowing that they were infected with COVID-19, can be estimated [3].

These serological tests are known to be in use in other countries to figure out how many people in their population are infected with the potentially deadly virus. For example, results from Spain’s final stage of a nationwide antibody study shows that 5.2% of Spain’s population has been exposed to the new virus as of July 6, 2020 [4], 0.07% (11 out 14,000) for Taiwan (0.05% after age correction for the entire population) from the adult patients who visited the Taipei Longminzung hospital in May and July, 2020 [5], one out of four Mexican citizens were found to have antibodies to the novel coronavirus infection with 70% being asymptotic from August to November, 2020 [6], and 6% of all regions of the UK and 13% in London alone of citizens recruited from 20 June to 13 July 2020 [7], 2.5% of Georgia, USA, and 3.2% of the general residents of Wuhan, China, have antibodies [8].

South Korea also recently released its third antibody titer test results for the Korea National Health and Nutrition Examination Survey (KNHANES) and military enlistment in addition to previous results found at Daum News (Seoul, 2020) [8]. Residents of 15 cities and provinces nationwide excluding Gwangju and Jeju participated in this third survey. In this study, a total of three showed positive antibody responses (2 of them even had neutralizing antibodies that neutralized the virus) out of 1,379 people who participated in the third round of the KNHANES from August 14 to October 31. The antibody formation rate calculated for undiagnosed confirmed cases excluding existing confirmed cases is 0.07% (1 out of 1,379). This is not significantly different from the results of the previous 1st survey (0.03%, 1 out of 3,553) and 2nd survey (0.07%, 1 out of 1,440). Also, a total of 25 people tested positive for 6,859 soldiers enlisted in September-October. Of these, 10 were confirmed patients, and the remaining 15 were infected by the local community. Therefore, the rate of formation of undiagnosed antibodies was 0.22% (15 out of 6,859), more than three times higher than 0.07% of the general public [8]. In total, 8,238 people were surveyed, giving a rate of antibody formation as 0.19%.

As stated previously in our article [3] on the same topic, the above-reported results in addition to being sparse, only captures sample proportion (point estimation) but does not provide its confidence interval which can be misleading to the general public, since confidence intervals give better interpretation to point estimation. Therefore, we report updated results for the point estimations along with proper interval estimations (95% confidence intervals) using asymptotic [10], exact [10,11], MidP [12], and Bayesian [13] statistical inferential methods already explained in detail in our previous article mentioned above.

## Results

Table 1 presents the 95% confidence intervals for antibody results using the sum of total samples from the first and the second survey and only KNHANES samples from the third survey. It also presents the 95% confidence intervals for the total population in South Korea. The first two columns show the methods and the next two columns the 95% confidence interval for antibody retention rate in the samples. The second and third columns represent the estimated 95% confidence interval of antibody carriers in the Korean population by multiplying the total number of Korean population (51,289,593 people, as of December 19, 2020 [14]) with the antibody ratio (the proportion of samples with neutralizing antibodies provided as confidence intervals [CIs]). Note that this estimation was derived from a simple random sampling assumption, while the antibody sample does not represent the total Korean population (Data: 3 [= 0 + 1 + 1 + 1] out of 5,874 [= 1,555 + 1,500 + 1,440 + 1,379].

From Table 1, Wald gives the minimum upper bound which is 55,906 whereas MidP provides the narrower confidence intervals among all types of confidence interval methods. Score CI from asymptomatic estimation methods and exact CI from exact estimation method are almost similar. Same goes for Likelihood ratio CI from asymptomatic estimation methods and Jeffrey’s CI from Bayesian estimation method.

Table 2 presents the 95% CIs of antibody results using the sum of samples of all cases (first, second, third [both KNHANES and military personnel]) and also for the total population in South Korea. The second and third columns show the methods whereas the next two columns show the 95% confidence intervals of antibody ratio for the total samples (Data: 18 [= 0 + 1 + 1 + 1 + 15] out of 12,733 [= 1,555 + 1,500 + 1,440 + 1,379 + 6,859]).

The MidP-value method gives the narrowest interval among exact estimation method for Tables 1, 2, and Supplementary Table 1. The Bayesian method using the uniform prior gives the narrower interval than the Bayesian method using Jeffrey’s prior. For asymptotic estimation, Wald method gives the narrowest interval for general and military personnel but when we consider all cases, likelihood ratio method gives the narrowest interval.

**Fig. 1.**
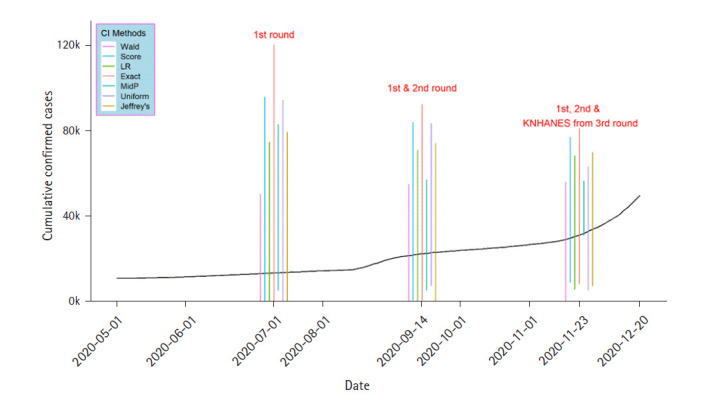
Confidence intervals (CIs) for the first to third antibody titer test results. The black line indicates the coronavirus disease 2019 cumulative confirmed cases. KN­HANES, Korea National Health and Nutrition Examination Survey.

As the sample size increases, the confidence interval becomes narrower; which indicates that more accurate estimation of antibody formation rate is possible (Fig. 1). Through an actual test, the lower bound can be replaced by the number of confirmed patients in the confidence interval. Among the upper bound, the smallest value provides a conservative interpretation while the largest value provides a more aggressive interpretation.

Antibody titer testing helps in (1) discovery of neutralizing antibodies if present in the population. Neutralizing mAbs promise an adjunct to vaccines and traditional drugs in the treatment of COVID-19 [1]. (2) It measures how far the infection has spread in the population. For example, Mexico’s results show that about 31 million people in Mexico already have COVID-19, much higher than the current official count of about 1,267,000 cumulative confirmed cases as of December 17, 2020 [6]. By subtracting a day’s cumulative number of confirmed cases from the smallest upper limit, the result is interpreted as the minimum number of COVID-19 cases that were infected but not confirmed. For South Korea, as of 00:00 on November 11, 2020, at least 69,524 (= 100,528–31,004) [15] people were infected but not confirmed, higher than the previous value of 32,602 as of September 15, 2020. This can be interpreted as having a high probability of cumulative infection.

South Korea’s antibody formation rate of 0.0014 (0.14%) which suggests that about 71,805 (= 0.0014 × 51,289,593) people in the population have already been exposed to COVID-19 virus as of November 23, 2020, is lower than for most foreign countries. For example, recently USA, Italy, and Sweden [16], Mexico (24.8%), Spain (5.2%), and UK (6%) have higher antibody rate but Taiwan has the lowest here of 0.07%. This demonstrates the adequacy and effectiveness of Taiwan's quarantine measures in mitigation and suppression of the virus. Although South Korea’s antibody titer result (0.14%) is lower than for most foreign countries, more positive cases were found among the young male people in their twenties (0.22%), three times that of the general public (0.051%) (Supplementary Table 1). This means that there are relatively many infected people among the younger generation but undiscovered since most younger age groups even if infected, are asymptomatic or mild. And given that they are active in social activities without receiving medical treatment or examination at a medical institution, the risk of spreading infection among the population is quite high. Therefore, this calls for the quarantine authorities’ need to strengthen quarantine managements for the early twenties in order to find hidden infected people in the population. However, the difference in these proportion between the young people and the general population is not statistically significant (p = 0.2491) by Fisher exact test.

## Figures and Tables

**Table 1.** 95% CIs for antibody results using the sum of total samples from the first and the second survey and only KNHANES samples from the third survey and for the total population in South Korea

Point estimation	Interval estimation					
	Method		95% CI (antibody retention rate)		Total population 95% CI (antibody carriers)	
			Lower	Upper	Lower	Upper
3/5,874 = 0.0005 (0.051%)	Asymptotic estimation	Wald	0.00000	0.00109	0.00	55,906
		Score	0.00017	0.00150	8,719	76,934
		Likelihood ratio	0.00011	0.00133	5,642	68,215
	Exact estimation	Exact	0.00016	0.00158	8,206	81,038
		MidP	0.00060	0.00110	30,774	56,419
	Bayesian estimation	Uniform	0.00010	0.00123	5,129	63,086
		Jeffrey’s	0.00014	0.00136	7,181	69,754

CI, confidence interval; KNHANES, Korea National Health and Nutrition Examination Survey.

**Table 2.** 95% CIs of antibody results for all three samples and for the total population in South Korea

Point estimation	Interval estimation					
	Method		95% CI (antibody retention rate)		Total population 95% CI (antibody carriers)	
			Lower	Upper	Lower	Upper
18/12733 = 0.0014 (0.14%)	Asymptotic estimation	Wald	0.00076	0.00206	38,980	106,169
		Score	0.00089	0.00223	45,648	114,376
		Likelihood ratio	0.00077	0.00197	39,493	100,528
	Exact estimation	Exact	0.00089	0.00224	45,648	114,889
		MidP	0.00110	0.00210	56,419	107,708
	Bayesian estimation	Uniform	0.00084	0.00213	43,083	109,247
		Jeffrey’s	0.00087	0.00218	44,622	111,811

CI, confidence interval.

## Supplementary Materials

Supplementary Table 1. Comparison of 95% CIs of antibody results using the sum of samples from general population and only samples form young population as well as total population in South Korea
