# Supplementary material for: Updated confidence intervals for the COVID-19 antibody retention rate in the Korean population
Source: Genomics Inform. 2020 Dec 24;18(4):e45. doi: 10.5808/GI.2020.18.4.e45 (PMC7808864; doi:10.5808/GI.2020.18.4.e45)
Supplement: Supplementary Table 1. — Comparison of 95% CIs of antibody results using the sum of samples from general population and only samples form young population as well as total population in South Korea [file gi-2020-18-4-e45-suppl.pdf]

Supplementary Materials

Supplementary Table 1. Comparison of 95% CIs of antibody results using the sum of samples from general population and only samples form young population as well as total population in South Korea

Method	Public				Young public (Military personnel)			
	95% CI (antibody retention rate)		Total population 95% CI (antibody carriers)		95% CI (antibody retention rate)		Total population 95% CI (antibody carriers)	
	Lower	Upper	Lower	Upper	Lower	Upper	Lower	Upper
Asymptotic estimation								
Wald	0.00000	0.00109	0.00	55,906	0.00108	0.00329	55,393	168,743
Score	0.00017	0.00150	8719	76,934	0.00133	0.00360	68,215	184,643
Likelihood ratio	0.00011	0.00133	5642	68,215	0.00123	0.00349	63,086	179,001
Exact estimation								
Exact	0.00016	0.00158	8206	81,038	0.00132	0.00362	67,702	185,668
MidP	0.00060	0.00110	30774	56,419	0.00160	0.00310	82,063	158,998
Bayesian estimation								
Uniform	0.00010	0.00123	5129	63,086	0.00122	0.00342	62,573	175,410
Jeffrey’s	0.00014	0.00136	7181	69,754	0.00128	0.00351	65,651	180,026

CI, confidence interval

From the above table, for both general and young population the MidP method gives the minimum upper bound which are 56,419 and 158,998 respectively. The MidP method also provides the narrowest confidence intervals among all types of confidence interval methods for young population.
